# Diagnostic and Therapeutic Indications for Endoscopic Ultrasound (EUS) in Patients with Pancreatic and Biliary Disease—Novel Interventional Procedures

**DOI:** 10.3390/curroncol29090488

**Published:** 2022-08-29

**Authors:** Manfred Prager, Elfi Prager, Christian Sebesta, Christian Sebesta

**Affiliations:** 1 Department of Surgery, Hietzing Clinic, Vienna Hospital Association (WIGEV), 1130 Vienna, Austria; 2 Department of Medicine I, Endoscopy, Wiener Neustadt Hospital, 2700 Wiener Neustadt, Austria; 3 ScienceCenter Donaustadt, 1220 Vienna, Austria; 4 Gastroenterology and Oncology Clinics of Donaustadt and Floridsdorf, Vienna Hospital Association (WIGEV), 1220 Vienna, Austria

**Keywords:** endoscopic ultrasound (EUS), pancreatic cancer, cancer surgery, pancreatic cysts, EUS-targeted biopsy

## Abstract

There is growing evidence supporting the substantial, essential and indispensable role of endoscopic ultrasound (EUS) as a key diagnostic armamentarium for upper GI oncologic surgery. Well described in countless publications, EUS holds that position in gastroenterological expert centers all over Europe. Despite its undisputable contributions to oncologic upper GI surgery, the availability of this technique at the expert level shows up in an irregular spread pattern. Endoscopic ultrasound’s primary use during the first few years after its creation was the detection of pancreatic cancer. From then on, EUS developed in different directions, becoming a diagnostic tool that increasingly better defines its status as a method of minimally invasive therapeutic applications and a useful addition to surgical options. As a result, several surgical interventions could even be replaced by ultrasound-targeted interventions. This process took place in just a few years and was made possible by technical development that sensibly combined high-resolution ultrasound with therapeutic endoscopy. The present article will serve to cover the most prevalent uses with supporting data considering the growing list of suggested indications for EUS while also examining cutting-edge initiatives that might soon become the standard of clinical practice. Endoscopic centers with high expertise are needed to train future experts in the growing field of EUS interventions.

## 1. Introduction

Oncology in general and surgical oncology of the upper abdomen, especially the diagnosis and therapy of pancreatic diseases, would today be unthinkable without endoscopic ultrasound (EUS). It has long played a vital role in the examination of organs in the upper abdomen, as reflected in international and national guidelines. This examination can evaluate for malignancies in the upper gastrointestinal tract, changes in the mediastinal lymph nodes, benign pancreatic lesions, pancreas malignancies, as well as submucosal tumors in the esophagus, stomach and duodenum [1,2]. EUS has gradually changed from a purely investigative diagnostic procedure to a minimally invasive solution for therapeutic interventions. The aim of this article is to describe the current status of EUS in oncology and in oncological surgery of the pancreas and bile ducts using essential study data and quoting from such sources. 

### Technical Requirements

In using high-frequency transducers to produce high-resolution ultrasound images, endoscopic ultrasound combines endoscopy and intraluminal ultrasound. During the early 1980s, transabdominal ultrasound procedures were the only option available to diagnose diseases of the biliary system and the pancreas. However, nowadays, abdominal ultrasound has gained much higher efficacy. In the differential diagnosis of obstructive jaundice, conventional ultrasound achieved similar results to those of MRCP (90% vs. 98%, respectively). Considering the validity of the diagnosis of pancreatic carcinoma, tumor size is a decisive criterium. The sensitivity ranges from 78% to 89%, respectively. The transabdominal diagnostic procedure is hampered by intestinal gas that causes limited visibility [3,4,5].

Two distinct types of EUS scopes are used:

Radial scope: The scanning plane is vertical to the axis of the scope; 360 degrees; currently almost exclusively used for staging and not suitable for interventions.

Linear scope: The scanning plane is parallel to the transducer; intervention tool; significantly longer learning curve due to the altered anatomical picture. Needles: 19, 20, 22, 25 gauge (G); depth of penetration up to 10 cm.

## 2. Training and Learning Curve

In several European countries and in the US, the use of endoscopic ultrasound is part of the curriculum for ‘high-level’ gastroenterologists. The learning curve is exceptionally long. To learn how to use EUS, excellent transabdominal sonographic knowledge is needed as well as mastery of the entire set of endoscopic tools. 

One of the most important issues arises from the limitations of the method. For the effectiveness of the EUS diagnostic site and the size of the lesions examined play a crucial role. 

A number of prospective studies prove that EUS performs with high sensitivity, particularly for lesions smaller than 2–3 cm compared with CT and MR [6].

In terms of localization, the rates of sensitivity differ from head to tail of the organ. The validity of the findings varies between 83−92% for the head over 79% down to 37–40% for the tail [7,8,9].

EUS instruction programs are available for skill-based medical training and are prescribed by professional associations. 

Historically, colonoscopy [10,11], ERCP [12] and EUS [13] went through similar development in terms of the number of supervised procedures that trainees need to absolve. Even if acquiring EUS skills is similar to acquiring colonoscopy and ERCP skills, assessing EUS skills is much more difficult. Clearly defined quality indicators apply for colonoscopies and ERCP: the cecum intubation rate [14] for colonoscopy and cannulation of the native papilla of Vater for ERCP [15]. Unfortunately, there is no clear quality indicator for EUS, but the competence of examiners obviously increases with the number of examinations carried out [16,17]. On that account, it is difficult to define a universal procedural criterion for EUS that would apply to all EUS procedures. Among other factors, this is also due to the varying characteristics of the many different indications for EUS (staging, biopsy, intervention). Naturally, EU fine needle biopsy (FNB) rates, EUS FNB sensitivity and the complication rate could be defined as potential skill quality indicators, but there are arguments against the use of these criteria [18]. One key argument is that there is no way to quickly assess these quality indicators directly after the examination itself, i.e., practicability in the assessment of skills is lacking. As early as 2015, Sachin Wani published a promising EUS evaluation system where he combined the cognitive evaluation of the findings, therapeutic ultrasound skills and other analytical elements in a text system for trainers, in order to enable them to assess as objectively as possible, the knowledge and skills of the gastroenterologists who practice the EUS [19].

However, the system will likely be difficult to implement in clinical practice.

## 3. Overview of Indications for EUS-Targeted Biopsy in the Upper GI Tract

- Solid tumors of the pancreas;
- Cystic tumors of the pancreas;
- Submucosal tumors (SMTs) in the esophagus, stomach and duodenum;
- Diffuse wall thickening of the esophagus and stomach;
- Esophageal malignancies;
- Gastric cancer;
- Mediastinal lesions;
- Extraluminal lymph nodes;
- Selected liver lesions.

The following section will focus on diagnostic and new therapeutic procedures in the case of pancreatic and biliary disease.

### 3.1. Pancreatic Malignancies—PDAC (Pancreatic Ductal Adenocarcinoma)

EUS was initially developed in the late 1980s and 1990s for pancreas tumor diagnosis and for the identification of papilla Vateri tumors and submucosal lesions in the upper gastrointestinal tract. It soon became clear that, in specific cases, this examination technique was superior to transabdominal ultrasound [2]. Right from the start, EUS exhibited a sensitivity of over 90% in the detection of malignant pancreatic tumors [20]. Up until then, pancreatic tumors were identified on the basis of abnormal lab results and abdominal ultrasound as well as CTs. However, the tumors identified using these procedures were usually in an advanced stage. For pancreatic cancer, contemporary studies suggest that EUS has a sensitivity of up to 99 percent, with malignant pancreatic tumors measuring between 2 and 3 cm. This clearly demonstrates its overwhelming superiority over all other tomography devices, including CT, transabdominal ultrasound and magnetic resonance [6,21]. This is primarily due to the possibility of moving the endoscopic-sonographic transducer to the immediate proximity of the lesion. Of course, EUS is not free of errors (for example, regarding the differentiation between early malignant and post-inflammatory lesions in the pancreas) and has its limitations regarding specificity in the diagnosis of pancreatic carcinoma. Diagnosis becomes more difficult when, in addition to pancreatitis, the pancreatic tissue displays heterogeneous morphology, so even seasoned examiners may overlook pancreatic cancer. On the other hand, there are studies that imply that EUS provides higher diagnostic accuracy than CT for pancreatic malignancy in chronic pancreatitis. There are criteria and predicting factors (hypoechoic pattern, pancreatic duct dilation, distal pancreatic atrophy) that help to differentiate benign from malignant lesions in patients with chronic pancreatitis. New developments in MR technology have produced a valuable supplementary diagnostic technique specifically for cases where malignancies are suspected, and difficult examination conditions reduce diagnostic reliability [22,23]. An MR provides greater connective tissue contrast than CT images, which, in the final analysis, allows varying pancreatic tissue masses to be differentiated more accurately [2,24]. Eleven percent of all PDACs are isodense, meaning they take up the same amount of contrast agent as the surrounding tissue, and this share increases to as much as 27 percent in the case of small-diameter tumors (≤2 cm). In this case, a combination of MR, dual-energy CT (with enhanced contrast) and EUS is helpful. EUS provides a differentiated view of pancreatic tissue morphology and remains one of the most accurate tools for the identification of unclear lesions. On that account, EUS is the method of choice for diagnosing and staging pancreatic adenocarcinoma [25]. It does not only have high specificity in detecting malignancies but (reversely) also serves as the primary means to exclude pancreatic cancer [21]. After a 24-month observation period, a group of patients who were referred to EUS due to abnormal CT findings in the pancreas and suspected adenocarcinoma did not develop any pancreatic malignancy, as predicted by EUS. This ultimately yields a negative predictive value (NPV) of 100 percent [21].

### 3.2. Cytology and Histology

Nowadays, combining EUS and fine needle aspiration cytology (FNA) or fine needle biopsy (FNB) is the standard everyday procedure. To be able to initiate adequate treatment, a lesion needs to be cytologically or histologically confirmed. In particular, when primary surgery is impossible, locally advanced tumors are unresectable a priori (locally advanced pancreatic ductal adenocarcinoma, LAPDAC), or neoadjuvant therapy is indicated in the case of a ‘borderline-resectable’ tumor. In retrospective analyses of EUS databases, EUS-FNB has been associated with a diagnostic precision of 89 percent for solid pancreatic tumors [24,26,27,28]. The possibility of obtaining biopsies of a suspected malignant pancreatic lesion directly during an imaging procedure has an immediate bearing on the medical management of the affected patients. Since only a minority of patients are eligible for curative therapy right away, obtaining cytological and histological material for diagnostic confirmation is absolutely necessary so that (possibly neoadjuvant) chemotherapy can be started or continued [26,27,28].

Lately, reports on cases of intra-abdominal tumor seeding have emerged, especially after EUS-FNA occurred. Although these complications seem to be extremely rare, the impact on the further faith of the patients may be tremendous. Safety measures such as shortening the puncture path, decreasing the number of punctures and adding needle sleeves could minimize the risk of this unwanted complication. Nevertheless, prospective controlled studies must be carried out to objectify the frequency of the phenomenon and its impact on survival [29].

### 3.3. On-Site Pathology

The likelihood of arriving at a diagnosis with high specificity is markedly improved by the attendance of on-site pathologists, especially when conventional biopsy needles are used for the purpose of aspiration cytology (FNA). A prompt assessment reduces the risk of completing an examination, although the tissue samples taken are unusable [30,31,32,33]. With the support of on-site pathologists, EUS-FNA biopsy can facilitate earlier diagnosis and possibly suggest an alternative diagnosis, thereby reducing patient mortality. Apparently, diagnosis is improved on account of the direct communication between pathologists and endoscopists and the possibility of sharing an endoscopic impression. Ever since new types of 19-, 22- and 25-gauge needles with ultra-sharp blades have found their way into routine practice as a means of obtaining histological and cytological material, the presence of on-site pathologists, whose attendance has often been requested (but very rarely approved in practice), has become even more expedient. As the technique is more refined, not only conventional cell aspiration (FNA) but also the collection of tissue using fine needle biopsy (FNB) and thus a more accurate diagnosis have become possible [24,25].

EUS is not only superior to conventional ultrasound or MR in certain cases when it comes to identifying and excluding pancreatic malignancies but also serves as a supplement to these non-invasive procedures in a meaningful way [6,21].

Even in cases of papilla Vateri adenoma and carcinoma, EUS can be used to correctly assess the invasiveness of the lesion and the endoscopic resectability where necessary.

## 4. Cystic Neoplasms of the Pancreas

### Pancreatic Cystic Lesion

The first step when confronted with cystic pancreatic lesions is to distinguish them from pseudocysts by differential diagnosis. In histopathological terms, pseudocysts frequently turn out to be ductal ectasia rather than a cyst or an actual cystic neoplasm of the pancreas. Pseudocyst diagnosis is usually assumed on the basis of anamnestic records, and a patient history consistent with pancreatitis usually points the way [34]. Biochemical analysis of the cyst content and imaging usually provide additional information. However, patients with cystic neoplasms occasionally also have concomitant pancreatitis or pancreatitis developing as a result of congestion induced by the neoplasm. Furthermore, patients with a pseudocyst do not necessarily always have a history that indicates previously occurring pancreatitis. Once pseudocysts have been excluded as a diagnosis, the type of cystic neoplasm of the pancreas must be determined.

Broadly speaking, there are two types of cystic neoplasms: mucinous cystic neoplasms, which include intraductal papillary mucinous neoplasms (IPMN) and mucinous cystic neoplasms (MCN) and serous cystic neoplasms (SCN). The paper of Sahani et al. [35] contains an overview of cystic lesions and neoplasms of the pancreas.

Patients with mucinous cysts, meaning patients with main duct (MD) IPMN and mixed type (MT) IPMN as well as MCN, undergo surgery if operability is possible, i.e., the cyst is resected.

After exclusion of any ‘worrisome’ or ‘high-risk features’ (which constitute a relative or absolute indication for surgery), imaging is performed in most branch duct (BD-)IPMN cases. Generally, the consensus policy should be adhered to [36].

MR and EUS imaging is usually performed on patients with SCN unless the diameter of the cyst exceeds 4 cm, tumor-associated symptoms present themselves or the cyst exhibits a high growth rate.

In cases of solid pseudopapillary tumors (SPNs), surgical resection is absolutely necessary [35,37,38].

## 5. EUS-FNB

There is no generally valid consensus policy for EUS-FNB indication in cases of cystic neoplasms of the pancreas.

EUS with or without FNB is not indicated if tomography provides a clear, reliable diagnosis. However, in case of doubt, an additional referral to EUS is key to ensuring a final diagnosis and personalized therapy because of the specific endosonomorphologic imaging provided and the cystic fluid analysis [39].

The high resolution and better image characteristics compared to CTs are a clear benefit. Endoscopic ultrasound makes sense if the CT-/MR-based diagnosis is uncertain, if cysts exhibit what are referred to as ‘worrisome features’ and if the malignancy diagnosis needs to be verified for high-risk patients ahead of surgery (comorbidity, age). Cyst fluid can be aspirated and analyzed, and grape-like nodes and small intracystic tumors can be biopsied and histologically/cytologically examined. Lymph node metastasis can be identified, biopsied and their relation to the primary tumor can be verified. Frequently (but not always reliably), vascular invasion can be established or excluded. A growing number of new biomarkers are being identified in cyst fluid that can be used to predict malignancy with increasing certainty [40]. In addition to analyzing cyst fluid to determine its biochemical and cytological composition and analyzing DNA in the cells found, the macroscopic assessment of cysts fluid can provide initial indicators early on: a highly viscous fluid, distinguishable on account of its behavior during aspiration into the biopsy syringe, is very likely to have a high mucin content and thus indicate IPMN or MCN. Even with a sensitivity of 73 percent and a specificity of 84 percent, the high CEA concentration in the cyst fluid is also indicative of a mucinous, unlike a non-mucinous, cystic neoplasm of the pancreas; a cut-off of 190 ng/mL is frequently cited in such cases [41]. Conversely, the CEA level in the cyst fluid is not predictive of the lesion’s malignancy.

## 6. The Most Important Diagnostic and Therapeutic Interventional Indications

### 6.1. Choledocholithiasis

Around 20 percent of patients with known cholelithiasis develop stones in the common bile duct. Identifying stones in the common bile duct continues to be a challenge because lab results and clinical manifestations can be unspecific [42]. Over many years, endoscopic ultrasound was tested in various studies for its ability to accurately diagnose choledocholithiasis. Endoscopic retrograde cholangiopancreatography (ERCP) is the method of choice. In contrast to CT examination and transabdominal ultrasound, ERCP has an accuracy of nearly 100 percent (CT, ultrasound: 50 percent); the sensitivity of MR cholangiopancreatography (MRCP) is almost identical. However, ERCP is an invasive method that, in the interventional setting, is associated with clinically significant (rarely lethal) complications [42,43]. On the other hand, ERCP is hardly used solely for diagnostic purposes nowadays but chiefly for therapeutic purposes, for instance, to remove stones from the common bile duct. Complications can occur in up to 11 percent of patients undergoing ERCP [43,44]. Numerous studies conducted in the 2000s compared the ability of EUS to verify or exclude choledocholithiasis to ERCP in combination with papillotomy and MR or with surgical exploration using interoperative cholangiography. All the studies showed that EUS could produce equivalent results, but ERCP remains the measure of all things when it comes to therapeutic intervention [42,44]. In a meta-analysis, 27 prospective cohort studies comparing EUS with ERCP, intraoperative cholangiography or surgical exploration were able to prove that EUS achieves 98 percent accuracy in choledocholithiasis diagnosis. This impressive level of accuracy is likely attributable to its high resolution of up to 0.1 mm, which outperforms ERCP or MRCP [42].

Some clinics continue to perform intraoperative cholangiography during laparoscopic cholecystectomy to check for patency of the biliary system. Choledocholithiasis is established in up to 15 percent of patients. However, up to 60 percent of the findings are false positives [43]. Given the sometimes remarkably high error rate of intraoperative cholangiography and the rate of complications with ERCP as a potential outcome of such a false positive diagnosis, a less invasive alternative would be attractive. EUS could have a potential role in the routine diagnostic algorithm prior to every ERCP. EUS is just as sensitive but considerably more specific than either ERCP or MRCP, particularly in the case of stones with a small diameter. However, the general use of EUS as a primary diagnostic tool has its limitations. Although EUS has significantly fewer complications than ERCP, a second session under sedation is required. Moreover, a very experienced endoscopist is needed for this examination in order to ensure the (above-mentioned) degree of accuracy. With the guidance and support of experts, this already straightforward task represents a benchmark for EUS training and can possibly also serve as quality criteria. However, these and other training objectives are currently only achieved at specialized centers.

### 6.2. Pancreatitis

#### Abscess, Pseudocyst, Walled-Off Necrosis (WON) Drainage

Pancreatic pseudocysts, involving abdominal pain, gastric outlet obstruction, early feeling of fullness, weight loss, icterus, infection or progressive increase in size, are general indications for interventions and thus represent the possibility of carrying out intervention including EUS [1].

Acute pancreatitis is frequently complicated by peripancreatic accumulation of inflammatory fluid. According to the revised Atlanta classification [45], acute peripancreatic fluid collections in cases of interstitial edematous pancreatitis are distinguished from acute necrotizing debris following intra- or peripancreatic retention in cases of necrotizing pancreatitis. Both these changes are described in the first four weeks of symptom onset. This is followed either by the development of pseudocysts after interstitial edematous pancreatitis or walled-off necrosis after necrotizing pancreatitis.

Most pancreatic pseudocysts heal spontaneously. Large and infected symptomatic pseudocysts must be drained. This is still the domain of the plastic stent. The use of an increasingly lumen-apposing metal stent (LAMS, e.g., Axios^TM^), may have additional benefits but is more expensive [46,47,48]. In recent times, experts tend to use LAMSs, which are easier to place, especially in the treatment of symptomatic pseudocysts. The principle of the procedure is to combine the LAMS along with an overlapping double pigtail stent for endoscopic transmural drainage of ‘pancreatic fluid collections (PFCs)’ with solid tissue parts. Compared to the procedure involving only the placement of a LAMS, drainage is more effective and requires fewer endoscopic follow-up interventions [49].

### 6.3. Walled-Off Necrosis

Plastic stents are often ineffective or have only little benefit because their narrow lumen is frequently occluded by viscous necrotic debris. With the recent development of fully covered lumen-apposing self-expandable metal stents (LAMS, see above), this issue has finally been resolved. LAMSs are also able to drain fluid or semifluid debris with high consistency and avoid leaks along the newly created drainage tract [50,51]. The saddle-shaped or double-T stent has double-walled ends in order to maintain the position on both sides in the stomach or the pseudocysts or the walled-off necrosis. The complete cover with silicon and the self-expandable radial forces prevent a leak along the newly formed canal. What is more, a LAMS can be removed. It can be implanted endoscopically/endosonographically without any radiological assistance, but having an imaging modality available in the event of complications is definitely an advantage [52]. The learning curve for this therapeutic intervention is decidedly shorter than for staging carried out for purely diagnostic purposes or the endosonographic morphological assessment of rare tumors of the biliopancreatic system.

There is a fierce debate that sets off the drainage of walled-off necrosis using multiple plastic stents against the treatment using lumen-apposing covered self-expandable stents with a large inside diameter (large-lumen LAMSs). The dispute has yet to be settled; prospective studies on this topic are underway around the world. Outside the scope of these trials, the empirical decision has been rendered in favor of LAMS. In final analysis, metal stents have also proved to be more cost-effective in treating walled-off necrosis: in terms of expenses, the shorter stay in hospital and considerably fewer repeat revision procedures are arguments in favor of the one-off purchase of an expensive stent. Haemorrhagic complications that may occur when using LAMS can be reduced if they are removed or replaced after a period of 3 to 4 weeks. This helps to avoid erosion of vessels in the gastric wall and the emergence of pseudoaneurysms [53,54,55]. Removal of the stent within this period is recommended in the guidelines for the management of necrotizing pancreatitis published by the European Society of Gastroenterology and Endoscopy [56].

### 6.4. Endoscopic Debridement in the Event of Walled-Off Necrosis

The above-described LAMSs can be used for repeated endoscopic debridement of retroperitoneal necrotic cavities.

In addition to facilitating spontaneous drainage into the stomach, the large diameter of LAMSs allows repeated endoscopic necrosectomy to be performed in peripancreatic necrotic cavities where clinically indicated. Such early endoscopic necrosectomy, which can be carried out at the time of transmural stent placement, helps to resolve the clinical presentation of infected walled-off necrosis at an early stage. At the same time, the number of endoscopic interventions is reduced [57]. Unlike access through surgery after percutaneous CT drainage followed by video-assisted retroperitoneal debridement (VARD), the EUS-guided drainage of infected pancreatic necrosis described above, followed by endoscopic necrosectomy if necessary, also leads to far fewer pancreatic fistulas and shorter hospitalization [58,59]. Compared to open invasive surgical necrosectomy, ultrasound-guided endoscopic necrosectomy is more successful because it is suitable to reduce mortality in cases of necrotizing pancreatitis (presumably on account of the low invasiveness of the procedure) [58,60].

### 6.5. EUS-Guided Biliary Drainage

EUS-guided transmural biliary drainage is an expedient emergency maneuver when ERCP is unable to ensure drainage [61]. In this procedure, the biliary system is accessed transgastrically via the bile ducts of the left liver lobe or transduodenally in order to drain the extrahepatic bile duct. Once access to the left hepatic bile duct or main bile duct has been established via the stomach or duodenum, a guidewire is inserted until it passes the papilla of Vater. Using the rendezvous technique, complete biliary drainage can be achieved in combination with ERCP. Alternatively, plastic or metal stents (either partly or fully covered) can be placed directly through the newly created access in order to drain the biliary system. These stents are placed either as antegrade transpapillary stents or as transluminal stents through choledochoduodenostomy or hepaticogastrostomy. [62] Depending on the localization of the malignant occlusion, either a transhepatic or a duodenal approach is preferable. Both have similar rates of technical success and carry a low risk of complication. Transgastric access to the intrahepatic biliary system enables biliary drainage even when gastric outlet stenosis is present or anatomy has been altered surgically (e.g., BII, Z. n. Whipple procedure or Y-Roux) [63].

Compared to transhepatic biliary drainage, EUS-guided biliary drainage causes less pain, has fewer side effects and requires less frequent reinterventions. Furthermore, the hospital stay is always shorter [64]. In a center with the required expertise, guided biliary drainage is the standard procedure when ERCP drainage is impossible. Transhepatic biliary drainage is thus no longer merely the treatment of choice after ERCP has failed. Very recent studies suggest that EUS-guided biliary drainage could become the first-line treatment in patients with malignant stenosis of the distal bile duct [65].

### 6.6. Cholecystitis

Percutaneous transhepatic drainage of the gallbladder is usually the treatment for high-risk patients with acute cholecystitis who are unfit to undergo cholecystectomy due to comorbidities. In recent years, EUS-guided drainage has become an alternative treatment method to create a fistula tract between the gallbladder and the stomach or the duodenum. Right after the development of the fully covered self-expandable lumen-apposing metal stents were these stents brought in as a minimally invasive endoscopic replacement procedure (instead of acute cholecystectomy) that entails only a minimized risk of bile leakage. Furthermore, meta-analysis has shown that, in the hands of experts, these procedures are reliable and involve a low rate of complications, while showing a success rate of over 90% [66,67].

There is also the option of combining the different procedures and implementing them metachronously: patients who have initially undergone percutaneous transhepatic gallbladder drainage and are unfit for surgical cholecystectomy in the follow-up can undergo conversion to transgastric EUS-guided (LAMS, AXIOS) internal drainage [68]. As soon as they have recuperated from their acute event, patients who underwent EUS-guided biliary drainage, finally can undergo laparoscopic cholecystectomy after a certain interval [69].

### 6.7. EUS-Guided Pancreatic Duct Drainage

Inaccessibility of the papilla of Vater and obstruction of the pancreatic duct by stones or strictures in patients with chronic pancreatitis as a consequence of pancreatic duct disconnection on account of necrotizing pancreatitis, following surgical procedures or in the event of pancreaticoenterostomy stricture, can theoretically be treated with EUS-guided pancreatic duct drainage. In these interventions, acceptable results were only achieved by a small and elite group of experts. Indications for EUS-guided pancreatic duct disobliteration are rare, and the procedure still appears to have a long way to go before it becomes part of any clinical routine outside specialized pancreas centers. For the indications specified in the guidelines issued by professional associations that have been tested in clinical routines over the years, stent implants are a useful and efficient alternative within the scope of ERCP [70].

### 6.8. EUS-Guided Gastroenteroanastomosis

Recent years have seen the development of new methods for the treatment of benign and malignant pyloric stenosis where a gastroenteroanastomosis is created using the EUS-guided approach. LAMSs were used in all cases to construct the anastomosis. From the EUS position in the stomach, the distal section of the duodenum or a jejunal loop can usually be drawn to the stomach wall through a puncture. For this purpose, the target lumen is punctured with a needle. This produces an anastomosis between the stomach and the duodenum or the stomach and the jejunum. To ensure that the puncture to access the small intestine is safe, the small intestine is filled with water. To do this, if in some way technically feasible despite the obstruction, an ultra-thin endoscope is inserted via the stenosis, and saline solution is injected into the small intestine via the endoscope. If there is any technical way of introducing a balloon via the existing structure, the balloon filled with fluid on the other side of the stenosis will also be recognized as the intraluminal target. A clinical evaluation of these techniques shows that EUS-guided gastroenteroanastomosis is similarly successful in clinical terms and just as safe as the application of a duodenal stent [71].

### 6.9. Biliary Access in Patients with Altered Anatomy: Bariatric Surgery

If ERCP is indicated following a gastric bypass (Y-Roux gastric bypass, omega loop), a connection between the gastric pouch and the remainder of the stomach can be created by implementing a LAMS with a particularly large lumen in an ultrasound-guided procedure. The ERCP can then be introduced via the stent that has been placed and then via the gastric remainder [72,73]. This EUS-guided gastro–gastric anastomosis and subsequent ERCP is far superior to ERCP double-balloon enteroscopy in terms of feasibility and the results (100 percent v 60 percent, *p* < 0.001) [74]. With this technique, connections between Y-Roux loops and other loops of the small intestine can even be created in patients with distal malignant obstruction.

### 6.10. Ablation Techniques—Therapeutic and Palliative Tumor Ablation Using a EUS-Guided Procedure

With the help of real-time imaging, EUS can be used to place needles or other tools directly into tumor masses. On this account, various types of destructive energy sources, radio-opaque markers (fiducials), anti-tumor agents and radioactive particles (seeds) can be administered [75]. ‘Fiducials’ are markers that can be placed using an ultrasound-guided procedure or around the tumor tissue and help to better administer radiotherapy in a more targeted manner [76,77]. Where patients are unfit for surgery, liver lesions can be treated via percutaneous tumor ablation using radio frequency waves or ethanol. EUS-guided ablation techniques using radio frequency waves and ethanol injections are implemented not only in patients with cystic and solid pancreatic tumors but also in patients with hepatic and adrenal tumors. [63] The endosonographically guided instillation of substances that target tumors plays a palliative role in the therapy of cystic neoplasms of the pancreas. This is how the chemotherapeutic Paclitaxel and alcohol or macrogol are instilled [63,78,79]. When it comes to the instillation of Paclitaxel in cystic neoplasms of the pancreas, remission was observed after lengthy observation periods in long-term studies [80].

For non-resectable pancreatic carcinoma, feasibility studies have shown that radio-frequency ablation and heating of the tissue to temperatures above 45 °C induce successful protein degradation and thus cause irreversible cell damage. These interventions work well from a technical perspective, but no benefit for overall survival has been established so far [81].

In clinical routine, the following local ablation procedures are widely used:

- Thermal ablation;
- Radiofrequency ablation;
- Photodynamic therapy;
- Microwave ablation;
- High-intensity focused ultrasound;
- Cryoablation;
- Neodymium-doped yttrium aluminium garnet.

Damage to the surrounding structures, including the duodenum and ductus choledochus, is an issue that all thermal ablation procedures in the pancreatic area have to face. In addition, ablation of the entire tumor may be impossible due to its dimensions. Despite these limitations, many studies have documented a growing interest in local ablation procedures. Thermal ablation is used to treat inoperable pancreatic carcinoma, neuroendocrine tumors of the pancreas and pancreatic metastases, and the applications have achieved promising results [82,83,84]. In the palliative setting, loco-regional ablation techniques entail lower morbidity, improved preservation of vital structures in the tumor’s surroundings and, last but not least, shorter hospital stays than surgical intervention. The general progress achieved, especially in terms of thermal ablation techniques, has triggered a process that will likely promote the application of these treatment methods via the endoscope [85,86]. The possibility of imaging vessels using duplex ultrasound at every stage of the EUS procedure is very helpful. For now, the ablation technique best adapted to EUS is radiofrequency ablation, where local temperatures between 60 and 100 °C are generated in the targeted tissue. This temperature causes irreversible cell damage, apoptosis and intentional coagulation necrosis in the affected tumor region [84,87].

### 6.11. Coeliac Plexus Block

EUS-guided coeliac plexus block or ganglia neurolysis and block is a procedure used to manage pain in palliative pancreas carcinoma patients. This palliative method for pain elimination or minimization can also be used in cases of chronic pancreatitis. Accordingly, its applications markedly reduce the need for traditional pain relievers.

In this procedure, a neurolytic agent (usually ethanol) is centrally injected into the base of the coeliac plexus or directly into the coeliac ganglia [88,89]. Coeliac ganglia can be identified reliably by using EUS and may be destroyed completely or partially by ethanol injection in a highly selective on target procedure. High-volume ethanol injections are well-suited to ensure diffuse, complete and irreversible coeliac neurolysis. Unidentified ganglia can also be treated this way [89]. Peri-interventional developments that may occasionally occur include worsening pain experience, diarrhoea and hypotension. Retroperitoneal haemorrhage, ischemia or abscess formation are extremely rare complications. Compared to coeliac plexus block, the combination of coeliac plexus ablation and primary tumor ablation using ethanol proved to be more effective in treating pain and even resulted in a slightly improved survival outcome [90].

### 6.12. EUS-Guided Fine Needle Tattooing (EUS-FNT) of Tumors

The development of laparoscopic methods has drastically changed the surgery of the biliopancreatic system. Whereas methods such as complete mobilization of the pancreatic body, tail and spleen used to be generally accepted for bimanual palpation to search for small pancreatic neuroendocrine tumors, they now belong to medical history.

However, minimally invasive surgical techniques require exact initial localization of the biochemically identified tumors. At the time of surgery, this localization must be demonstrably visualized in a laparoscopic image.

For this reason, EUS-guided fine needle tattooing (EUS-FNT) was developed out of a number of historical precursor procedures.

In 2002, EUS-FNT was applied for the first time for the purpose of intraoperative identification of a pancreatic lesion during surgery [91]. During the procedure, an insulinoma measuring 20 mm × 5 mm was localized at the transition from the body to the tail of the pancreas, exactly between the splenic vein and splenic artery. For marking, India ink was injected into the tumor through a fine needle aspiration tool. Surgery was performed by laparotomy, and on account of the unfavorable position for surgery, pancreatic tail resection in combination with splenectomy was necessary.

As laparoscopic resection became possible for smaller parts of the pancreatic body and tail for the removal of solid and cystic lesions in recent years, accurate pre-operative localization became all the more important. The often-misleading occurrence of retroperitoneal peripancreatic fatty tissue can make the performance of intraoperative ultrasound considerably more complicated. Nevertheless, experts are able to reliably pinpoint the exact location of the lesion in 60 to 90 percent of the cases [92,93]. The consequences of unsuccessful intraoperative localization are reoperation and/or residual tumors as well as tumor growth in the parts of the pancreas left in place by mistake. For these cases, EUS-FNT is an absolutely indispensable procedure in the range of interventions offered by a center of pancreatic surgery.

## 7. Summary and Conclusions

Originally devised as an efficient tool to stage tumors of the upper abdomen (esophagus, stomach, duodenum and pancreas), endoscopic ultrasound has moved on to become a highly developed intervention tool. The immense significance of EUS lies in its versatility as a staging tool and as a means to obtain tissue for the accurate classification of lesions and thus for initial prognostic assessment. The second and even more important impact of the method is its meaning for the choice of different and graded as well as individualized therapeutic approaches. Particularly when it comes to diseases (also inflammatory) of the pancreas and the biliary system, EUS has charted a continuous upward trajectory.

The diagnosis of pancreatic and biliary neoplasms would simply be unthinkable without EUS on account of its diverse and constantly growing therapeutic applications. After establishing EUS as an unquestionable useful diagnostic tool, therapeutic applications are currently experiencing an upgrade in possibilities and safety. Thanks to EUS, crucial decisions regarding the retention or removal of organs or the performance or non-performance of endoscopic resection can be made, especially when the involvement of the lymph nodes can be established by imaging. A biopsy of the lymph nodes rounds out the information provided by classical tomographic morphology findings. This has direct implications for the therapeutic approach and the prognosis of the disease.

In addition to thermal ablation and the injection of radiological markers in support of radiotherapy or direct tumor-destructive agents, interventions such as biliodigestive drainage and connections such as gastroenteroanastomosis can also be performed by newly developed technologies (see above).

This paper intends to provide an overview of the currently available diagnostic and therapeutic potentials of using endosonographic procedures as a minimally invasive option to supplement and enhance the treatment of various diseases of the upper gastrointestinal tract, especially the pancreas and the bile ducts.

It will become necessary in the near future to direct attention specifically to structured EUS training.

Adopting the training culture used in the Anglo-American and Asian medical systems will further improve the benefits of the procedures described and expand the range of therapeutic options available to include less invasive methods with manageable complication rates.

The authors believe that EUS should be integrated, using an evidence-based approach, into the diagnostic and therapeutic spectrum of multidisciplinary pancreas centers as soon as possible. The literature has provided convincing evidence of its usefulness in diagnostics and as a valuable addition to the therapeutic armamentarium [6,94,95].

Any technical and methodical extension to the range of EUS applications and the interventional possibilities of EUS should undergo prospective scientific evaluation in order to define its importance in the full spectrum of interventions and to evaluate and objectify the limits of the method at the same time.
